# Supplementary material for: Nerve injury induces pain hypersensitivity and anxiety-related behaviours and is associated with amygdala activation in male mice
Source: PLoS One. 2025 May 7;20(5):e0323203. doi: 10.1371/journal.pone.0323203 (PMC12058149; doi:10.1371/journal.pone.0323203)
Supplement: Attachment — Submitted filename: Rev Plos one.docx [file pone.0323203.s004.docx]

The manuscript by Tong and colleagues provides valuable insights into neuropathic pain-induced anxiety-like behavior in rodents, offering a potential animal model to study the neural basis of pain and the comorbid negative affective states. However, the manuscript lacks clarity, and additional experiments would be beneficial to enhance the impact of the research. Below are my general suggestions for improving the quality and novelty of the manuscript to be considered for publication in PLOS ONE:

1. **English Language and Clarity**: The manuscript’s use of English needs significant improvement. The writing is often difficult to follow, and, in some instances, it presents conflicting ideas within the same paragraph. For example, in lines 132 and 133, it is stated that SNI both changes and remains unaffected in terms of distance traveled within the same sentence.
2. **Inclusion of Female Mice**: The study would be more impactful and generate greater interest if female mice were also included. Previous literature (e.g., Burek, 2022; Lorente et al., 2024) has demonstrated a sex-dependent effect of pain on anxiety-like behaviors. In fact, male mice sometimes fail to exhibit increased anxiety-like behavior in the presence of pain.
3. **Introduction and Literature Context**: The introduction should be better grounded in existing literature. The selection of the amygdala as the nucleus of interest is relevant to the field, but providing more detailed data from previously published studies would strengthen the rationale for focusing on the amygdala in this context.
4. **Timing of Testing**: The timing of testing is not specified, which is crucial as rodent behavior can be influenced by the time of day in relation to the light cycle. Please specify the testing times, considering the light cycle.
5. **TBST Composition**: More details on the composition of TBST should be provided, particularly concerning the concentration of the detergent used.
6. **Discussion of Contradictory Data**: The discussion should address contradictory findings that have been published in the existing literature.
7. **Analysis of PrL, PVT, and PAG**: While the roles of the PrL, PVT, and PAG are of significant interest, it is difficult to draw conclusions about their involvement without analyzing these areas. If the researchers still have access to the brains of the animals used, including an analysis of these regions would be fundamental to substantiate claims regarding their roles.
8. **Minor Comments**: Abbreviations should be used consistently throughout the manuscript, and the English should be further refined.
