# Supplementary material for: Nerve injury induces pain hypersensitivity and anxiety-related behaviours and is associated with amygdala activation in male mice
Source: PLoS One. 2025 May 7;20(5):e0323203. doi: 10.1371/journal.pone.0323203 (PMC12058149; doi:10.1371/journal.pone.0323203)
Supplement: Attachment — Submitted filename: Response to Reviewers.docx [file pone.0323203.s006.docx]

Dear editor and referees,

We are grateful to you for providing us an opportunity to revise our manuscript entitled “Nerve injury induces pain hypersensitivity and anxiety-related behaviors via amygdala activation in male mice” (Manuscript ID: PONE-D-24-27569).

We deeply appreciate the constructive comments of the reviewing editor and reviewers. We believe your suggestions and comments have helped to improve the quality of the manuscript significantly and that it will now be deemed suitable for publication. We have revised our manuscript according to the reviewers’ comments and provided point-by-point responses to all their concerns. The referenced changes have been made in the revised manuscript and are highlighted in red.

We thank you for your consideration of our manuscript and look forward to your favorable decision.

Yours sincerely,

Yuanyuan Wu,

[runnaway@126.com](mailto:runnaway@126.com)

Reviewer #1: The manuscript by Tong and colleagues provides valuable insights into neuropathic pain-induced anxiety-like behavior in rodents, offering a potential animal model to study the neural basis of pain and the comorbid negative affective states. However, the manuscript lacks clarity, and additional experiments would be beneficial to enhance the impact of the research. Below are my general suggestions for improving the quality and novelty of the manuscript to be considered for publication in PLOS ONE:

1. English Language and Clarity: The manuscript’s use of English needs significant improvement. The writing is often difficult to follow, and, in some instances, it presents conflicting ideas within the same paragraph. For example, in lines 132 and 133, it is stated that SNI both changes and remains unaffected in terms of distance traveled within the same sentence.

Response:

We thank you for the suggestion. We regret the mistake and have corrected the inaccurate description (page 5, line 158). In addition, we further checked the language of the article to make the expression more accurate.

2. Inclusion of Female Mice: The study would be more impactful and generate greater interest if female mice were also included. Previous literature (e.g., Burek, 2022; Lorente et al., 2024) has demonstrated a sex-dependent effect of pain on anxiety-like behaviors. In fact, male mice sometimes fail to exhibit increased anxiety-like behavior in the presence of pain.

Response:

Thanks for your valuable suggestion. Before we started this experiment, we did have the stereotype that male mice might be more stable in behavior. We also followed the previous research paradigm involving only male mice [1-3]. In the follow-up experiments, we will also use female mice as our research subjects to further investigate this sex-dependent effect. Thanks again for your valuable comments.

[1] Xu Y, Zhu X, Chen Y, et al. Electroacupuncture alleviates mechanical allodynia and anxiety-like behaviors induced by chronic neuropathic pain via regulating rostral anterior cingulate cortex-dorsal raphe nucleus neural circuit. CNS Neurosci Ther. 2023;29(12):4043-4058. doi:10.1111/cns.14328

[2] Chen Y, Tong S, Xu Y, et al. Involvement of basolateral amygdala-rostral anterior cingulate cortex in mechanical allodynia and anxiety-like behaviors and potential mechanisms of electroacupuncture. CNS Neurosci Ther. 2024;30(9):e70035. doi:10.1111/cns.70035

[3] Wu Y, Chen Y, Xu Y, et al. Proteomic Analysis of the Amygdala Reveals Dynamic Changes in Glutamate Transporter-1 During Progression of Complete Freund's Adjuvant-Induced Pain Aversion. Mol Neurobiol. 2023;60(12):7166-7184. doi:10.1007/s12035-023-03415-7

3. Introduction and Literature Context: The introduction should be better grounded in existing literature. The selection of the amygdala as the nucleus of interest is relevant to the field, but providing more detailed data from previously published studies would strengthen the rationale for focusing on the amygdala in this context.

Response：

Thanks for your valuable advice. We admit that the introduction in our previous manuscript is too simple. In response to your suggestion, we have revised the introduction to include more detailed data and information from previously published studies. We have also included additional references to relevant studies. These additions aim to strengthen the rationale for our focus on the amygdala and to provide a clearer context for our study within the existing body of literature. (page 2-3, line 32-78).

4. Timing of Testing: The timing of testing is not specified, which is crucial as rodent behavior can be influenced by the time of day in relation to the light cycle. Please specify the testing times, considering the light cycle.

Response：

Thanks for your advice. We would like to clarify that all testing was conducted during the light phase of the animals' diurnal cycle, specifically between 9 AM and 3 PM. This time frame was chosen to minimize any potential effects of the light cycle on the behavioral outcomes. We have revised and supplemented the first draft accordingly to more accurately describe our experimental methods (page 4, line128-129).

5. TBST Composition: More details on the composition of TBST should be provided, particularly concerning the concentration of the detergent used.

Response:

We apologize for not including this information in the initial submission and have now updated our methods section to include the detailed composition of TBST (page 5, line 137-138). The revised details are as follows:

TBST buffer composition:

Tris-buffered saline (TBS): 20 mM Tris-HCl, 150 mM NaCl, pH 7.4

Tween 20: 0.1% (v/v)

Thank you once again for your valuable feedback.

6. Discussion of Contradictory Data: The discussion should address contradictory findings that have been published in the existing literature.

Response:

Thank you for your suggestion. In response to your comment, we have revised the discussion section of our manuscript to include an analysis of the contradictory data published in the literature. We have carefully reviewed relevant studies and provided our interpretation of how these findings relate to our own results. We have revised the discussion section to address and discuss any contradictory findings reported in the existing literature, ensuring a comprehensive analysis of the topic (page 6, line 199-204). Thank you once again for your valuable feedback and suggestions.

7. Analysis of PrL, PVT, and PAG: While the roles of the PrL, PVT, and PAG are of significant interest, it is difficult to draw conclusions about their involvement without analyzing these areas. If the researchers still have access to the brains of the animals used, including an analysis of these regions would be fundamental to substantiate claims regarding their roles.

Response:

Thank you for highlighting the importance of analyzing the PrL, PVT, and PAG regions. We acknowledge that our current data do not directly address the involvement of these areas. Unfortunately, we no longer have access to the brains of the animals used in this study. However, we plan to include an analysis of these regions in our future studies to further investigate their roles and substantiate our claims.

8. Minor Comments: Abbreviations should be used consistently throughout the manuscript, and the English should be further refined.

Response:

Thanks for the suggestion. We have reviewed the manuscript carefully and ensured that all abbreviations are used consistently throughout the text. As for the refinement of the English language, we understand that clear and concise writing is essential for effective communication of our research findings. We have taken your comments into consideration and have revised the manuscript to improve the clarity and precision of our language. We have also proofread the text carefully to eliminate any grammatical errors or awkward phrasing. We are grateful for your suggestions and believe that they have helped us to improve the quality of our manuscript.

Reviewer #2: In this manuscript Wu et al, assess neuropathic pain impact on hyperalgesia and anxiety-like behaviors using VonFrey and Elevated Plus Maze/Open Field Tests, respectively.

The authors provide an extremely brief introduction lacking proper context or rigor to present their investigations. This is followed by a brief presentation of the methods used, a very succinct description of the results, and a discussion in which many elements are disconnected from the data shown in the manuscript.

Overall:
1. the experiments shown by the authors have been repeated on multiple occasions in the literature

Response:

Thank you for your valuable advice. We acknowledge the significance of these previous studies and their contributions to the field. Our intention in including these experiments was not to claim novelty, but rather to provide a robust foundation for our subsequent analyses and conclusions. By repeating the experiments, we aimed to confirm the consistency and reproducibility of the results, which strengthens the overall confidence in the findings. We believe that our study adds value by providing a fresh perspective and contributing to the ongoing dialogue in the field. And this is also the basis of our next research project. We appreciate your valuable feedback and have made the necessary adjustments to our manuscript.

2. there is a lack of description for the Sham control group - did the controls received a Sham surgery or no procedure at all

Response:

Thank you for raising the concern regarding the description of the Sham control group. We apologize for any lack of clarity in our manuscript. For the sham group, mice underwent the same procedure but without nerve ligation or transection. We have now added a more detailed description of the Sham surgery in the Methods section to ensure transparency and reproducibility of our results (page 4, line 95-96).

3. the CFos experiment is not associated with specific acute painful event, which might be fair but at least needs to be discussed

Response：

Thank you very much for your valuable feedback. We acknowledge that our study focuses on chronic pain and, as such, the design does not directly link c-Fos expression to a particular acute painful stimulus. However, we believe that this approach is justified in the context of chronic pain research, as it allows us to investigate neuronal activation patterns associated with sustained pain states rather than transient, acute events ^[1]^. Our future studies will utiliz more direct measures of neuronal activity, such as calcium imaging or electrophysiology, will be essential for elucidating the precise mechanisms underlying amygdala involvement in pain and anxiety. We also added a discussion of the limitations of C-Fos experiments (page 6, line 212-215).
[1] Xu Y, Zhu X, Chen Y, et al. Electroacupuncture alleviates mechanical allodynia and anxiety-like behaviors induced by chronic neuropathic pain via regulating rostral anterior cingulate cortex-dorsal raphe nucleus neural circuit. CNS Neurosci Ther. 2023;29(12):4043-4058. doi:10.1111/cns.14328

4. cFos analysis is presented as number of Fos positive neurons. the authors should consider a density of Fos positive per mm3 at least

Response:

Thank you for your suggestion. We appreciate your point that presenting the data as the number of Fos-positive neurons alone may not fully capture the underlying biology. To address this, we recalculated the density of c-Fos per square millimeter in the BLA and CeA across different sections (Fig 3B). As shown below：

5. the "positive correlation with the distance from Bregma" is nebulous and needs to be discussed in terms of rostro-caudal involvement of BLA/CeA in pain if needs to be presented.

Response:

Thank you for the valuable suggestion. We understand that the presentation may have been nebulous. We have clarified the anatomical significance of Bregma as a reference point in neuroscientific research and explained how the distance from Bregma can serve as a proxy for the rostro-caudal axis within the brain. We have delved deeper into the rostro-caudal distribution of BLA and CeA, highlighting the regional differences in their involvement in pain modulation. To address this, we have revised the manuscript to include a discussion on the rostro-caudal involvement of the BLA and CeA in pain regulation (page 5-6, line171-175). We believe that these revisions have significantly improved the clarity and depth of our discussion, and we are grateful for your guidance in helping us refine our manuscript.
