# Supplementary material for: Nerve injury induces pain hypersensitivity and anxiety-related behaviours and is associated with amygdala activation in male mice
Source: PLoS One. 2025 May 7;20(5):e0323203. doi: 10.1371/journal.pone.0323203 (PMC12058149; doi:10.1371/journal.pone.0323203)
Supplement: Attachment — Submitted filename: Response_to_reviewers_auresp_2.docx [file pone.0323203.s007.docx]

Dear editor and referees,

We are grateful to you for providing us an opportunity to revise our manuscript entitled “Nerve injury induces pain hypersensitivity and anxiety-related behaviors via amygdala activation in male mice” (Manuscript ID: PONE-D-24-27569R1).

We are writing to submit our revised manuscript for the second round of major revisions, incorporating all the suggestions and comments provided by both the reviewing editor and the reviewers. We deeply appreciate the constructive feedback and the time taken to review our manuscript. Your insights and guidance have been crucial in improving the quality and clarity of our work. We are confident that the manuscript, having undergone these significant changes, now meets the standards for publication.

We have carefully addressed each of the reviewer's concerns and made the necessary revisions to the manuscript. We have provided point-by-point responses to all the comments, highlighting the changes made in the revised manuscript in red for easy identification. We believe that these changes have greatly enhanced the readability and scientific validity of our work.

We sincerely hope that our revised manuscript will now be deemed suitable for publication in your esteemed journal. We are committed to providing any additional information or making further revisions as needed to ensure the manuscript meets your requirements.

Yours sincerely,

Yuanyuan Wu,

[runnaway@126.com](mailto:runnaway@126.com)

Reviewer #1: Although the authors have made improvements to the manuscript, some concerns remain only partially addressed. I believe these issues need to be fully resolved to ensure the manuscript's quality. Additionally, a more in-depth analysis of existing data and a refined discussion are essential.

Major Concerns:

1. English Language: the manuscript’s use of English still requires further improvement, particularly in terms of readability and clarity.

Response:

Thank you for your feedback on our manuscript. We acknowledge that the readability and clarity of the English could be improved. We thoroughly reviewed the manuscript to ensure that each point was conveyed in a logical and concise manner. This will help to improve the overall readability of the text.

2. Inclusion of Female Mice: if including female mice is not feasible, the authors must provide a more thorough discussion of sex differences by referencing data from other studies. They should also explicitly acknowledge that the exclusion of female mice is a limitation of this study. The justification provided—reliance on previous studies—no longer holds, as studies now often include female mice.

Response:

Thank you for your constructive suggestion. We fully acknowledge that the exclusion of female mice is a limitation of our current work. In response to your comments, we revised the manuscript to include a more comprehensive discussion of sex differences, referencing relevant data from recent studies that have included female mice. We understand that the reliance on previous studies, which may have excluded female mice, is no longer sufficient justification for our current approach. We acknowledge this limitation in the revised manuscript and emphasize the importance of future studies to address this gap in the literature. Thank you again for your valuable feedback. (page 6-7, line 224-235)

3. Testing Time and Light Cycle Effects: while the authors state that testing occurred between 9 a.m. and 3 p.m. to avoid light cycle effects, they have not provided data to substantiate this claim. To my knowledge, there are no studies proving that testing within this range minimizes light cycle effects. Importantly, behavioral testing conducted during the light phase (inactive phase) is less optimal, as it may yield different conclusions compared to testing during the active (dark) phase. This should be addressed in the discussion and compared with data from other studies. Furthermore, the exact timing of the light/dark cycle (lights on/off) should be reported.

Response：

Thank you for bringing to our attention the concerns regarding the testing time and potential light cycle effects on our study results. We would like to clarify that our behavioral testing was conducted in a dark room with wall lights, which was specifically designed to minimize any external light influences. We are sorry that we did not make this explicit in our previous manuscript. The testing room was equipped with blackout curtains and light-tight doors to ensure that no ambient light could penetrate during the experiments. Therefore, despite the fact that testing occurred between 9 a.m. and 3 p.m., the animals were exposed to consistent dimly lit conditions throughout the testing period. We understand that the timing of behavioral tests relative to the animals' light/dark cycle is an important consideration. However, in our study, the dark room conditions allowed us to create a controlled environment that was independent of the external light cycle. This allowed us to perform the tests at a consistent time each day without concern for light cycle effects. To address this point, we made the corresponding supplement in the method on the specific conditions of our testing environment. Thank you again for your thoughtful comments and suggestions. (page 4, line 130-133)

4. Discussion: while the discussion has improved, further refinement is needed. It should include a detailed examination of sex differences, the timing of testing, and a deeper comparison with existing literature.

Response：

Thank you for your constructive feedback on our manuscript's discussion section. Regarding the need for a detailed examination of sex differences, we acknowledge that this is an important consideration in our study. We referenced relevant data from recent literature that have included female mice. In the revised discussion, we will provide a more thorough analysis of any observed sex-specific effects and discuss their potential implications for our findings. (page 6-7, line 224-235)

Concerning the timing of testing, we understand that this can be a critical factor in behavioral studies. Our tests were conducted in a controlled environment (as previously mentioned, in a dark room to minimize light cycle effects). It is worth mentioning that our laboratory has developed relatively mature testing protocols for both the elevated plus maze and open field tests, and the current experiment follows the traditional operational paradigms established in our lab ^[1-5]^. Taking these into consideration, we have added specific supplements in the Methods section of the manuscript and have refrained from elaborating excessively on these points in the Discussion section.

We made these refinements to the discussion section promptly and ensure that the revised manuscript provides a comprehensive and nuanced analysis of our results in the context of current research.

Thank you once again for your valuable input.

[1] Wang YJ, Zan GY, Xu C, et al. The claustrum-prelimbic cortex circuit through dynorphin/κ-opioid receptor signaling underlies depression-like behaviors associated with social stress etiology. Nat Commun. 2023;14(1):7903. Published 2023 Nov 30. doi:10.1038/s41467-023-43636-x

[2] Chen Y, Tong S, Xu Y, et al. Involvement of basolateral amygdala-rostral anterior cingulate cortex in mechanical allodynia and anxiety-like behaviors and potential mechanisms of electroacupuncture. CNS Neurosci Ther. 2024;30(9):e70035. doi:10.1111/cns.70035

[3] Xie Y, Shen Z, Zhu X, et al. Infralimbic-basolateral amygdala circuit associated with depression-like not anxiety-like behaviors induced by chronic neuropathic pain and the antidepressant effects of electroacupuncture. Brain Res Bull. 2024;218:111092. doi:10.1016/j.brainresbull.2024.111092

[4] Zhu X, Zhang C, Hu Y, et al. Modulation of Comorbid Chronic Neuropathic Pain and Anxiety-Like Behaviors by Glutamatergic Neurons in the Ventrolateral Periaqueductal Gray and the Analgesic and Anxiolytic Effects of Electroacupuncture. eNeuro. 2024;11(8):ENEURO.0454-23.2024. Published 2024 Aug 28. doi:10.1523/ENEURO.0454-23.2024

[5] Wu Z, Shen Z, Xu Y, et al. A neural circuit associated with anxiety-like behaviors induced by chronic inflammatory pain and the anxiolytic effects of electroacupuncture. CNS Neurosci Ther. 2024;30(4):e14520. doi:10.1111/cns.14520

5. Statistical Analysis: the explanation of statistical methods is insufficient. Although a t-test may be acceptable for behavioral analysis with two groups, the manuscript does not indicate whether normality of the data was tested. Additionally, t-values are missing from the results section. For spatial analysis, a t-test is inappropriate. An ANOVA is necessary to compare conditions (SNI vs. sham) across different bregma distances.

Response：

Thank you for bringing these points to our attention. Regarding the normality testing, we acknowledge that we did not explicitly mention it in the manuscript. The normality of the distribution of continuous variables was assessed using the Shapiro–Wilk normality test. We added this information in the revised Methods section to ensure clarity. We also apologized for the oversight in not reporting the t-values in the Results section. We included these values in the revised manuscript to provide a complete picture of the statistical analysis. For the spatial analysis, we agree that a t-test may not be the most appropriate method for comparing conditions across different bregma distances. We conducted an ANOVA to analyze these data and reported the results in the revised manuscript. (page 5, line 152-160)

Minor Comments:

1. c-Fos Assumptions:
c-Fos is an immediate early gene whose expression increases shortly after neuronal activation. It is induced through calcium-dependent signaling pathways in response to excitatory input. While c-Fos levels may rise due to neuronal activation associated with injury or pain, it is not strictly indicative of nociception. The manuscript should avoid making this assumption.

Response：

Thank you for highlighting the important distinction between c-Fos expression and nociception. We fully agree that c-Fos is an immediate early gene whose expression can increase in response to neuronal activation associated with various stimuli, including injury or pain, but it is not a direct indicator of nociception. We will revise the manuscript to clarify this point and avoid making any assumptions that link c-Fos expression directly to nociception. Instead, we will emphasize that c-Fos expression is a marker of neuronal activation and should be interpreted in the context of the experimental paradigm and other available data. Thank you for your valuable feedback, and we appreciate your time and effort in reviewing our manuscript. (page 2, line 18; page 3, line 75)

Reviewer #2: I would like to thank the authors for taking into account my comments on their previous submission.
While the authors have answered some of my comments there are still some details left unresolved
1) The figure 2 still shows number of CFos neurons while the authors have shown these results as CFos+ cells/mm2. The comment previously made applied to all data using CFos

Response:

Thank you for bringing this inconsistency to our attention. You are correct that Figure 2 displays the number of c-Fos neurons, whereas we have presented the results elsewhere in the manuscript as c-Fos+ cells per square millimeter (c-Fos+ cells/mm²). To address this issue, we will revise Figure 2 to ensure that the units are consistent with the rest of the data presented. As shown below：

2) while there is a very brief discussion about the rostro-caudal axis data presented in Figure 3, it would be a good idea to compare the CFOs+ density in SNI versus Sham animals. This would provide a better picture of the pattern of activity in CeA/BLA induced by pain. Assuming of course that this dat comes from the same tissue as the one presented in Fig2 this should not be a complex analysis to run/add to the manuscript.

Response:

Thank you for your valuable suggestion. Comparing the cfos+ density between SNI and Sham animals in the CeA/BLA is indeed a promising idea. We have already conducted immunofluorescence staining statistics on the brain slices from the existing sham group. As shown below：

Thank you once again for your valuable suggestions.
