# Supplementary material for: Nerve injury induces pain hypersensitivity and anxiety-related behaviours and is associated with amygdala activation in male mice
Source: PLoS One. 2025 May 7;20(5):e0323203. doi: 10.1371/journal.pone.0323203 (PMC12058149; doi:10.1371/journal.pone.0323203)
Supplement: Attachment — Submitted filename: Response_to_reviewers_auresp_3.docx [file pone.0323203.s008.docx]

Reply to Reviewers
Manuscript ID: PONE-D-24-27569R2
Title: Nerve injury induces pain hypersensitivity and anxiety-related behaviours and is associated with amygdala activation in male mice

Dear Editor and Reviewers,

Thank you for your valuable feedback and the opportunity to revise our manuscript. We sincerely appreciate the time and effort dedicated by the reviewers to improve the quality of our work. We have carefully addressed all comments and revised the manuscript accordingly. Below are our point-by-point responses to the reviewers' suggestions.

We sincerely hope that our revised manuscript will now be deemed suitable for publication in your esteemed journal. We are committed to providing any additional information or making further revisions as needed to ensure the manuscript meets your requirements.

Yours sincerely,

Yuanyuan Wu,

[runnaway@126.com](mailto:runnaway@126.com)

Reviewer #3:

Comment #1: The title is not correct. You saw the effects are ASSOCIATED with amygdalar activation, not VIA amygdala. You don´t know if other brain areas were also strongly activated. So, please change the title to: "Nerve injury induces pain hypersensitivity and anxiety-related behaviours and is associated with amygdala activation in male mice"

Response: We sincerely appreciate your careful reading and constructive suggestion. We fully agree that the term "via amygdala activation" in the original title could imply a causal or exclusive mechanism, which is beyond the scope of our current findings. As suggested, we have revised the title to "Nerve injury induces pain hypersensitivity and anxiety-related behaviours and is associated with amygdala activation in male mice" to more accurately reflect the correlational nature of our observations. This change has been implemented in the revised manuscript (page 1 and 2, title section).

Other typos/mistakes to be corrected:

1. - line 37: "... experience accompanying egative" - "negative" is missing an "n"

Response: Thank you for your meticulous attention to detail. This typographical error has been corrected to "negative" in line 37 of the revised manuscript (page 3, line 29 ). We have also conducted a full proofread of the text to ensure no similar errors remain.

2. - lines 63-68: this sentence has 6 lines. Please break it in two sentences

Response: Thank you for identifying this overly complex sentence. We have restructured the original sentence (now lines 60-68 in the revised manuscript, page 4) to improve readability and logical flow. The revised text reads:

The amygdala, as a structurally complex and functionally diverse brain region, exhibits distinct anatomical structures and functional subdivisions across different dimensions. Through in-depth exploration, its fine architecture can be analyzed from multiple perspectives, which delineate the amygdala's internal organization. These multifaceted investigations further provide critical insights into its involvement in higher cognitive functions, particularly emotional processing, memory consolidation, and social behavior.

Additionally, we have reviewed other long sentences in the manuscript to ensure clarity and conciseness.

3. - Please improve the English of the sentence: "However, following the neuropathic pain induced by SNI, how rostro-caudal involvement of BLA and CeA remains to be unclear."

Response: We appreciate your suggestion to enhance clarity. The original sentence has been revised to improve grammatical structure and precision. The updated version in lines 66-68 (page 4) now reads:

"However, the differential involvement of BLA and CeA along their rostrocaudal axis in SNI-induced neuropathic pain remain to be fully elucidated."

4. - line 89 "...Consent to Participate" - this is not a human study.

Response: We sincerely apologise for this oversight. As this is an animal study, the phrase "Consent to Participate" has been removed from the Ethics Statement in line 89 (page 5).

5. - lines 158-160: the sentence "respectively. mixed repeated ANOVA was used to campare the espression of c-Fos across different bregma distance. A significance level of p < 0.05 was considered statistically significant." has several typos (". mixed", "campare", "espression" - correct them.

Response: We deeply appreciate your meticulous proofreading. The errors have been corrected as follows in lines 168-169 (page 8, Methods section):

". mixed" → ". Mixed".

"campare" → "compare".

"espression" → "expression".

6. - line 383: "(C) T The c-Fos expression" - there is an extra "T"

Response: Thank you for catching this formatting error. The redundant "T" in the subfigure label of Figure 4C has been removed (now line 429, page 17).
